# Does breastfeeding increase the risk of early childhood caries (ECC)? A systematic review

**DOI:** 10.1007/s40368-025-01051-4

**Published:** 2025-05-30

**Authors:** F. Alexaki, M. Kostopoulou, K. Koleventi, N. N. Lygidakis

**Affiliations:** 1 Aristotle University of Thessaloniki, Thessaloniki, Greece; 2 Private Paediatric Dental Clinic, Thessaloniki, Greece; 3 Air Force Hospital and Private Paediatric Dental Clinic, Athens, Greece; 4 Private Paediatric Dental Clinic, Athens, Greece

**Keywords:** Breastfeeding, Early childhood caries, ECC, Dental caries, Paediatric dentistry, Pediatric dentistry

## Abstract

**Purpose:**

To review the current evidence on the association of breastfeeding during the first years of life with the development of Early Childhood Caries (ECC**).**

**Materials and methods:**

A systematic review of literature was conducted in June 2019 and again in March 2024 at the following Databases: Pub Med, Scopus, Cochrane Library, Science Direct, Google Scholar, for studies reporting on children aged up to 71 months investigating breastfeeding duration/cessation and presence of caries at the examination. Prospective Cohort studies were included in the review. The systematic review was conducted according to PRISMA statement guidelines.

**Results:**

Of the 4894 papers identified, the data extraction protocol led to 8 studies for further review. Assessment of Risk of Bias was made using the ROBINS-E tool. Six studies were characterized as high risk of bias, one characterized with some concerns and one with low risk of bias. Breastfeeding for 6–12 months has a protective effect against ECC. There is no association between breastfeeding and ECC for the ages 12–24 months; however, depending on the frequency or when combined with increased sugar consumption, it can have an impact on dental caries prevalence. Beyond 24 months, breastfeeding was associated with increased ECC prevalence.

**Conclusions:**

Based on the studies included in this review and within their limitations, breastfeeding up to 2 years of age does not increase ECC risk, but after 2 years of age breastfeeding is associated with increased risk of ECC.

**Trial registration number:**

The protocol for this systematic review was registered on PROSPERO (CRD42020179773).

**Supplementary Information:**

The online version contains supplementary material available at 10.1007/s40368-025-01051-4.

## Introduction

Dental caries is one of the most common conditions affecting the general health of children (Petersen et al. 2005). In the United Kingdom, from 1973 to 2013, the caries prevalence decreased from 72 to 41% in 5 years, and from 97 to 46% in 15 years (Murray et al. 2015). In the United States, from 2002 to 2012, caries declined among children and adolescents from 54.1% to 45.2% (Kumar et al. 2015). However, despite a global decrease in the prevalence of caries the last 30 years (1990-2019), there is a significant increase in the caries burden, mainly driven by the rise in case numbers in certain populations and geographical areas (WHO 2022). In contrast, the prevalence of dental caries in the primary dentition of children under 6 years of age in Saudi Arabia has reached 84%, and by the age of 9 years, the prevalence has reached 94% (Al Agili 2013).

Early Childhood Caries (ECC) is defined as the presence of one or more decayed (non-cavitated or cavitated lesions), missing or filled (due to caries) surfaces, in any primary tooth of a child under 6 years of age (Pitts et al. 2019). Unfortunately, ECC remains a global problem, as was clearly indicated by a recent meta-analysis of cross sectional studies using the WHO criteria showing the prevalence of ECC to be 48% [95% CI 42,52] (Uribe et al. 2021). Additionally although there are several methodological issues in the relevant studies, it appears that there is an increase of ECC in the last 20 years. (Tinanoff et al. 2019).

World Health Organisation guidelines on breast feeding (BF) recommend its exclusive practice for the first 6 months with complementary breastfeeding up to 2 years of age or beyond (WHO 2023). The same approach for exclusive initially and complementary later up to 2 years breast feeding, is also implemented by both American and the European Academy of Pediatrics (Meek and Noble 2022; EAPD 2022). However, the American Academy of Pediatric Dentistry having evaluated the conflicting evidence and the possibility of causal effect between BF and ECC to the date of its policy making, recommends supporting a mother’s decision to breastfeed but avoiding ad libitum breastfeeding after tooth eruption (AAPD 2023).

The effect of breastfeeding on caries development is to this date a controversial issue. Breast milk, in contrast to formula, contains breast-specific Lactobacilli and substances, including human casein and secretory IgA, which inhibit the growth and adhesion of cariogenic bacteria, particularly Streptococci (Danielsson Niemi et al. 2009; Holgerson et al. 2013). Moreover, the phosphoproteins in milk inhibit enamel dissolution and lactose is not fermented in the same degree as other sugars (Tinanoff 2005). However, in a longitudinal investigation, a significant decline over time was observed in the levels of phosphate and calcium in breast milk that help protect tooth enamel (Greer et al. 1982). It has also been reported that human milk was significantly more cariogenic than cow’s milk probably because of its lower mineral content and higher level of lactose (Bowen & Lawrence 2005), but exhibited lower cariogenicity than infant formula or sucrose. The high concentration of lactose found in human milk has the potential to reduce the pH of dental plaque leading to dental caries (Prabhakar et al. 2010). Breast milk has superior nutritional composition and bioavailability; however, sugars provide approximately 40% of the energy in mature breast milk. Mature breast milk contains more sugars than bovine milk, approximately 7% compared with 4.8%. Breast milk is also significantly lower in calcium and phosphate (which protect against dental caries) compared to bovine milk (Poskitt & Stewart 2017).

There is inconsistency in the literature in whether breastfeeding significantly reduces dental plaque pH. Laboratory studies report that human breast milk can reduce dental plaque pH and cause greater dissolution of the enamel when compared with bovine milk (Rugg-Gunn et al. 2009). However, an in vivo experiment of plaque pH measurements of caries free and ECC children’s biofilms when exposed to both human milk and 10% sucrose solution, concluded that BF did not provoke a decrease in biofilm Ph irrespective of children’s caries status, whereas sucrose did (Neves et al. 2016). The authors attributed the outcome to an increase in salivary flow during the infant's suckling movements, which helps prevent the acidogenicity of milk lactose and, consequently, a drop in the biofilm's pH. However, this potential effect is likely lower than that of infant formula (Peres et al. 2008).

Frequent feeding will increase cariogenic potential as will nocturnal feeding due to decreased salivary flow during sleep (Nakayama & Mori 2015). On the other hand, a protective role of breastfeeding in the first year of life has been reported, possibly associated with less sugar consumption and delayed use of the bottle among children who are breastfed (Hong et al. 2014; Nirunsittirat et al. 2016).

Several studies have reported no association between breastfeeding and ECC prevalence (Iida et al. 2007; Kumarihamy et al. 2011; Mohebbi et al. 2008; Nunes et al. 2012; Roberts et al. 1993; Rosenblatt & Zarzar, 2004; Schroth et al. 2013), while others find a positive correlation between long duration BF and ECC (Chaffee et al. 2014; K Peres et al. 2017; Shrestha et al. 2024; Tanaka & Miyake 2012), nocturnal BF and ECC (Nakayama & Mori 2015; van Palenstein Helderman et al. 2006).

The aim of the present systematic review of the literature is to establish whether prolonged breastfeeding (beyond 24 months) can be associated with the prevalence of Early Childhood Caries (ECC).

## Materials and methods

The Preferred Reporting Items for Systematic Reviews and Meta-Analysis tool (PRISMA STATEMENT) was used as a guideline in the present systematic review. The review was registered in PROSPERO (CRD42020179773) before starting the data collection.

### Information sources and research strategies

The research question for this systematic review was conducted using PICO characteristics (Population, Intervention, Control, Outcome), as follows:

P—Population: Children 0–71 months. The cut off age of 71 months was used according to the definition of AAPD for Early Childhood Caries (AAPD 2008). 

I—Intervention: Breastfeeding continuing at 24 months or beyond. The cut off point of 24 months was chosen according to the recomendations of World Health Organisation for breastfeeding (WHO 2023). 

C—Control: Breastfeeding for less than 24 months.

O—Outcome: Association with Early Childhood Caries (ECC) assessed by dmft or dmfs index.

Research of relevant published studies was conducted using the following databases: Pub Med, Scopus, Science Direct, Cochrane Library, Google scholar. Grey literature and bibliographies of published articles were also searched to identify studies not identified previously. The initial research was conducted in June 2019 and a new search was conducted in March 2024.

The following keywords were used for breastfeeding studies:

(breastfeeding OR breast feeding) AND milk AND (caries OR decay OR ECC OR early childhood caries) AND (tooth OR teeth)—PubMed

(breastfeeding OR breast feeding) AND milk AND (caries OR decay OR ECC OR early childhood caries) AND (tooth OR teeth)—Scopus.

(breastfeeding OR breast feeding) AND milk AND (caries OR decay OR ECC OR early childhood caries) AND (tooth OR teeth)—Science Direct.

(breastfeeding OR breast feeding) AND milk AND (caries OR decay OR ECC OR early childhood caries) AND (tooth OR teeth)—Cochrane Library.

(breastfeeding OR breast feeding) AND milk AND (caries OR decay OR ECC OR early childhood caries) AND (tooth OR teeth)—Google Scholar.

The full search strategy is presented in the Supplementary material.

### Study inclusion and exclusion criteria

- Prospective Cohort Studies conducted in children 0–71 months were included in the present systematic review.
- Only English literature was included.
- Only studies investigating the effect of breastfeeding on Early Childhood Caries.
- Studies where intervention group was: children breastfed for 24 or more months and control group was: children breastfed for less than 24 months.

Exclusion criteria used when evaluating titles and abstracts are as follows: studies irrelevant to the subject of the study, cost–benefit analysis, Unsupported opinion of experts, Editor’s choice, Interviews, commentaries, Book’s -conference’s abstracts, animal studies, in-vitro studies, cross-sectional surveys, studies without an appropriate control group control, case reports or reports of cases, case–control observational studies, retrospective cohort studies, no English abstract, studies investigating dental pathologies other than ECC, studies reporting on infants with syndromes, studies reporting on children older than 71 months, and studies without reporting on the nutrition (breastfeeding). Table 1 presents the excluded studies and the reasons they were excluded.

**Table 1:** List of excluded articles and the rationale behind

Reasons for exclusion (For articles with more than one reason only one reason will be given)	Authors and year	Num of articles
Irrelevant population (i.e., wrong control group)	Kramer et al., 2007; Bowen et al., 2005; Suprabha et al., 2023	3
Irrelevant to the subject	Aimutis et al., 2012; Colombo et al., 2017; Yost et al., 2008; Prowse et al., 2014; Tang et al., 2013; Folayan et al., 2007; Meyer et al., 2018; Ripa et al. 1978; Neves et al., 2015; Schroth et al., 2015; Brecher et al., 2015; Hallas et al., 2011; Mohamed et al., 2018; Basir et al., 2017; Dye et al., 2004; Khodke et al., 2023; Carillo et al., 2021; de Souza et al., 2021; Kateeb et al., 2023	19
Irrelevant study design (i.e., in vitro)	Nakayama et al., 2015; Gomez et al., 1999; Uyttendaele et al., 1998; Majorana et al. 2014; Iida et al. 2007; Tanaka et al., 2012; Diley et al., 1980; Marrs et al., 2011; Makhdoom et al., 2015; Nishide et al., 2018; Rezvi et al., 2016; Ivančević et al., 2016; Petti et al., 2000; Achmad et al., 2018; Kakanur et al., 2017; de Carvalho et al., 2006; Sonis et al., 1997; Gussy et al., 2006; Ramos et al., 1999; Perera et al. 2014; Harmatiuk et al., 2016; Moura et al., 2006; Okawa et al., 2011; Turton et al., 2016; Alshehri et al., 2015; Bernabe et al. 2017; Erickson et al., 1999; Schroth et al. 2013; Setiawati et al., 2017; Jimenez et al., 2018; Panchanadikar et al., 2022; Bulut et al., 2022; Park et al., 2022; Suparattanapong et al., 2021; Chiao et a., 2021; Yadav et al., 2022; Al-Haj et al., 2021; GeethaPriya et al., 2023; Chanpumet al., 2020; Barjatya et al., 2020.; Feldens et al., 2018	41
Not in English	Degano et al., 1993	1
Not Found	Nakayama et al., 2022	1
Total		65

### Study selection

Titles and/or abstracts of the initial search were reviewed independently by two authors (MK, KK). Included studies were assessed independently by both authors. Any discrepancies were resolved by discussion and if that was not possible, by a third author (FA).

### Data extraction

Data was extracted by two reviewers in forms designed for this research. Each study had the following information extracted: publication details (authors, year of publication, design, location of study) sample characteristics (sample size, age of children at examination, breastfeeding duration) and outcome.

### Risk of bias assessment and certainty of evidence

The risk of bias of the included articles was evaluated by two independent reviewers (FA,

NNL) using the risk of bias in non-randomized studies of exposure (ROBINS-E) tool (Higgins et al. 2024) according to specific guidelines for observational cohort studies. Differences occurring during evaluation were discussed and resolved in consultation with a third reviewer (KK). Moreover, the certainty of current evidence was rated using the Grading of Recommendations Assessment, Development, and Evaluation (GRADE) criteria: risk of bias, indirectness, imprecision, inconsistency and publication bias. Two review authors (FA, NNL) independently judged the evidence certainty (high, moderate, low, or very low) and resolved disagreements by discussion with a third reviewer (KK).

## Results

### Search results

**Fig. 1:**
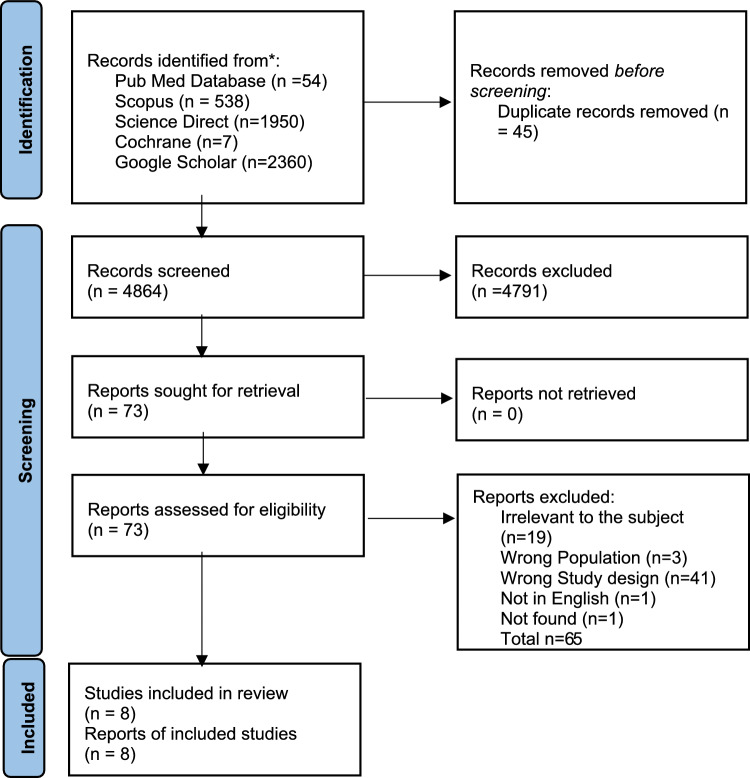
PRISMA flow diagram

In the search for breastfeeding articles, a total of 4909 articles were found. Exclusion of duplicates left 4864 articles to be evaluated. After Title–Abstract screening 4791 articles were excluded and 73 studies remain. From those, 65 were excluded (Table 1). Evaluation according to the eligibility criteria performed to the abstracts and full texts of the remaining articles, resulted in 8 articles to be reviewed (Fig. 1).

### Data synthesis

The main characteristics of the included articles were presented descriptively in a summary table with rows representing each article and columns representing the general characteristic (Table 2).

**Table 2:** Study characteristics of all included studies (n = 8)

Article	Subjects, n	Study design	Intervention (exposure)	Control	Outcome measure	Results
Birungi et al. 2017; Uganda	0–5 years children, n = 417	Prospective cohort study	Breastfeeding		ECC (dmft)	Exclusive breastfeeding for 6 months had a protective causal effect on ECC IRR(95% CI) = 1.01(0.981.04)
Chaffee et al. 2014**;** Porto Alegre, Brazil	0–3-year-old children n = 715	Birth cohort study	Breastfeeding 24 months or beyond	Breastfeeding 6–23 months	S-ECC (dmft)	Breastfeeding 24 months or beyond, particularly in frequent was associated with S-ECC
Hong et al. 2014; Iowa, USA	0–5-year-old children n = 509	Prospective cohort study	Breastfeeding duration < 6 months	Breastfeeding duration > / = 6 months	ECC (dfs)	Breastfeeding beyond 6 months seems to reduce the risk of carries p = 0.005
Nirunsittirat et al. 2016; Khon Kaen, Thailand	0–4-year-old children n = 556	Prospective cohort study	Breastfeeding duration 6–11 months, > 18 months	Breastfeeding < 6 months	ECC (dmfs)	Breastfeeding 6–11 months was a protective factor against ECC, prolonged breastfeeding was not associated with ECC RR = 0.45, 95% CI (0.22,0.90) *p* value = 0,02
Abanto et al. 2022; Amazon, Brazil	0–2-year-old children n = 800	Prospective cohort study	Breastfeeding duration 1223 months, > 24 months	Brestfeeding < 12 months	S-ECC (dmf-t)	The effect of prolonged breastfeeding on the increased risk of dental caries was slightly mediated by sugar consumption Bf < 12 months PR = 1/Bf 12– 24 months PR (95%CI) = 2.13 (1.46–3.11) Bf > 24m PR (95%CI) = 3.21 (2.12– 4.87) p < 0.001
Devenish et al. 2020; Australia	0–2/3-year-old children n = 1831	Prospective cohort study	Breastfeeding 1–6months, 6–12 months, > 12 months	Breastfeeding 0–1 month	ECC (dmft)	No independent association between breastfeeding beyond 1 y of age and ECC (PR 1.42, 95% CI 0.85, 2.38)
Feldens et al., 2010**;** Brazil	0–4 years n = 500	Randomized Trial	Home consultation about dietary habits (enforcement for exclusive breastfeeding up to 6 months and continue breastfeeding > 6 months)	No consultation	ECC–SECC (dmfs)	**ECC:** Intervention group: RR (95% CI) = 0.78 (0.65–0.93) Control group: RR = 1 *p* value = 0.004 **S-ECC:** Intervention group RR (95% CI) = 0.68 (0.50–0.92) Control group RR = 1.0 *p* value = 0.010
Perres et al. 2017**;** Brazil	0–5-year-old children n = 1303	Prospective cohort study	Breastfeeding duration 12–23 months, > / = 24 months	Breastfeeding duration 0–12 months	S-ECC (dmfs)	Breastfeeding 12–23 months does not have statistically significant difference in carries occurrence in comparison with breastfeeding up to 12 months < 12 months
						RR = 1,12–23 months RR = 1 For Bf > 24 months there is impact on carries RR = 2,4 (1,7–3,3)

### Study characteristics

All the trials included in the review were Prospective Cohort Studies published between 20,102,022 (Abanto et al. 2023; Birungi et al. 2017; Chaffee et al. 2014; Devenish et al. 2020; Hong et al. 2014; Nirunsittirat et al. 2016; Peres et al. 2017; Feldens et al. 2010). All studies reported the age of the children (0–71 months), sample size (ranging from 417 to 1831), and study duration at least 5 years. The study group was children that breastfed for 24 months or beyond and the control group was children breastfed up to 24 months. At the age of 5 years (71 months), an oral examination evaluating the prevalence of dental caries using dmft score as outcome, was performed. Table 2 summarizes the results of the studies.

### Risk of bias assessment

**Fig. 2:**
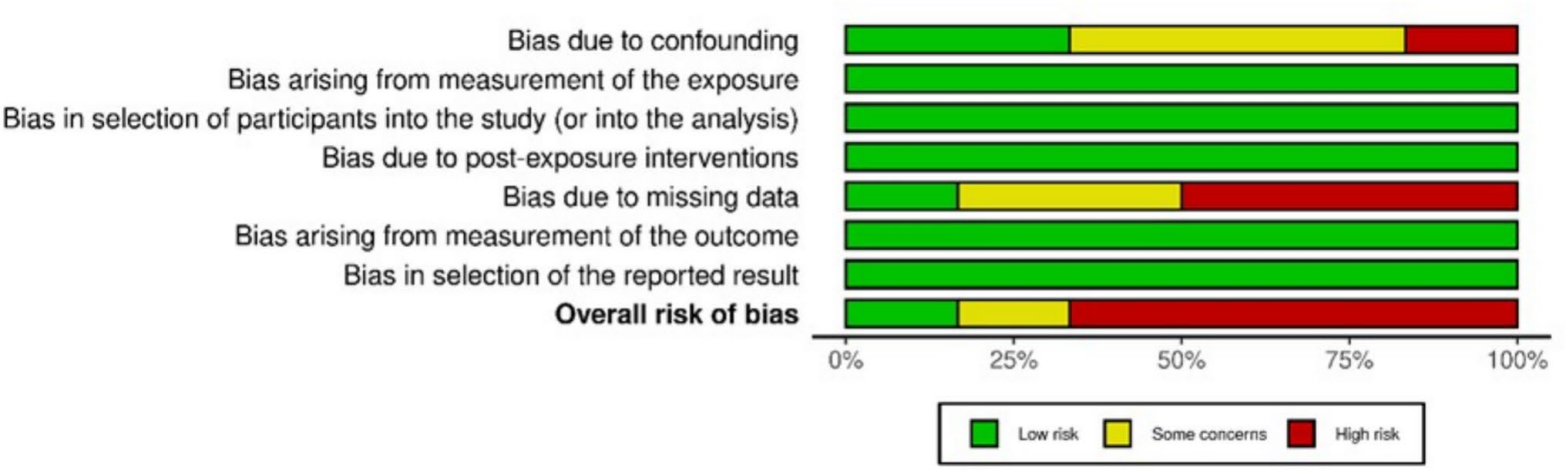
Overall risk of bias of the included studies assessed with the ROBINS-E tool. ROBINS-E, risk of bias in non-randomized studies of exposure

In total, six studies were assessed to be of overall high risk of bias. Two of them were rated as high risk of bias in the preliminary phase of ROBINS-E tool due to no controlling for confounding (Feldens et al. 2010) and inappropriate measurement of exposure and outcome (Hong et al. 2014). The remaining four (Abanto et al. 2023; Chaffee et al. 2014; Nirunsittirat et al. 2016) were assessed at the extended stage containing the seven domains. Figure 2 shows the risk of bias assessment for each domain separately and the overall assessment as a percentage of the included articles at the extended stage.

**Fig. 3:**
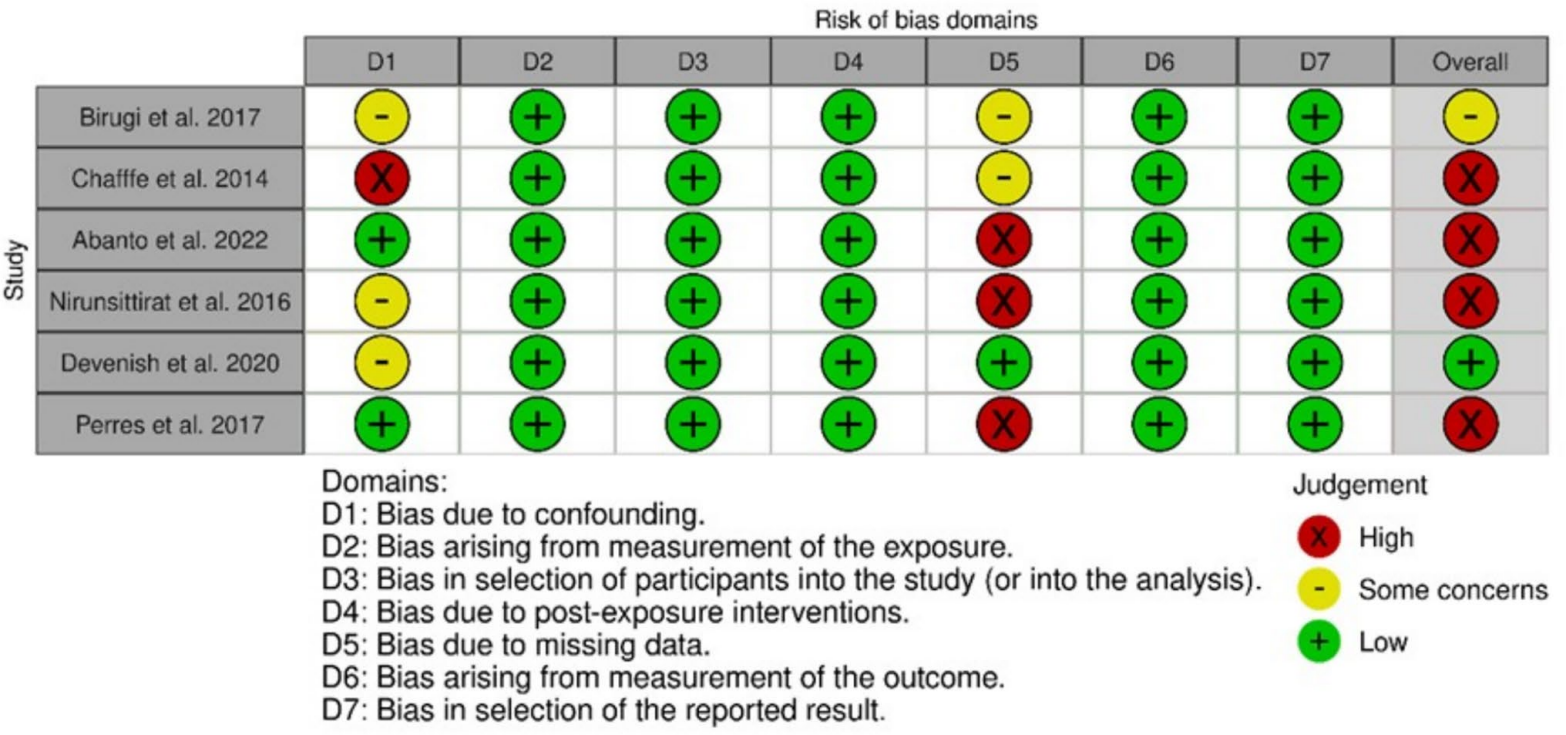
Risk of bias of each study for each domain and overall assessed with the ROBINS-E tool. ROBINS-E, risk of bias in non-randomized studies of exposure

Figure 3 contains the ratings for the individual domains for each included study. One study (Chaffee et al. 2014) was judged at high risk of bias, because they did not take into account the sugar consumption as a confounder (Domain 1). Sugar consumption is considered to be as one of the important risk factors and confounder for ECC alongside with the breastfeeding. Regarding the domain for the selection of participants into the study (Domain 3), in most studies follow-up began after the start of the exposure (breastfeeding), at 3 or 6 or 12 months from birth. However, this fact did not introduce any bias, because breastfeeding seemed to have a.

long latency period and, also, the first teeth tend to appear after 3 months of children’s age. Finally, three studies (Abanto et al. 2023; Nirunsittirat et al. 2016; K. G. Peres et al. 2017) were rated as high risk of bias due to no information or the analysis used for the missing data (Domain 5).

### Certainty of evidence

Table 3 provides details of the certainty of evidence assessment. The certainty of evidence was judged as low. The criteria for downgrading the quality of evidence included the following factors: (1) risk of bias: 7/8 of the included studies were assessed as having "some concern" or "high risk; (2) indirectness: in half of the studies, evaluation of breastfeeding as a risk factor for ECC was not the primary endpoint, but was associated among other factor; and (3) inconsistency: participants included in each study were either 0–2 y.o., 0–3 y.o, 0–4 y.o. or 0–5 y.o. ECC outcome was defined either as dmft or dmfs and the exposure variable breastfeeding had different categories among studies. Regarding the imprecision, the total number of patients included in all studies was above 400. The CIs of the estimates were narrow but, in some studies, the CIs were not reported. For this reason, the evidence was judged to have borderline imprecision. Finally, the presence of publication bias was not suspected.

**Table 3:**
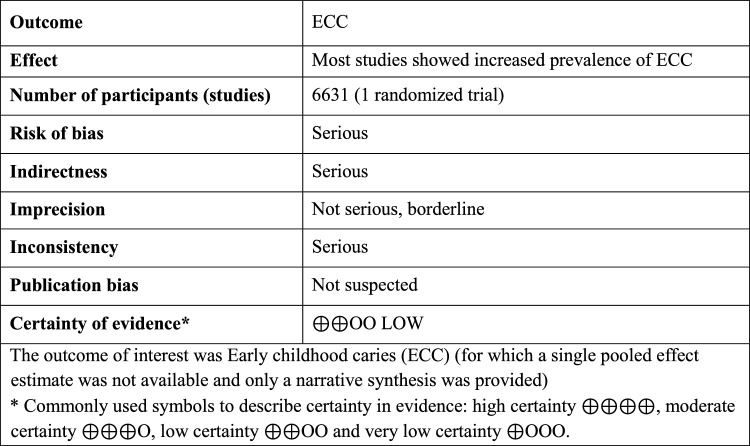
Summary of GRADE evidence

## Discussion

This systematic review highlights that breastfeeding has a protective effect against early childhood caries (ECC) during infancy (6–11 months). However, prolonged breastfeeding beyond 24 months is consistently associated with an increased prevalence of ECC. The findings indicate that breastfeeding and its association with ECC are strongly influenced by additional factors, such as nocturnal feeding, sugar consumption, and oral hygiene practices.

On one hand, several studies support the protective effect of breastfeeding in infancy. Clinical trials by Birungi et al. (2017) and Liang et al. (2014) found that exclusive breastfeeding for the first 6 months reduced the risk of ECC, with no significant association between breastfeeding duration and caries. Similarly, Feldens et al. (2010) demonstrated that exclusive breastfeeding beyond 6 months, combined with delayed sugar introduction, lowered ECC prevalence. Branger et al. (2019), Tham et al. (2015), and Shrestha et al. (2024) also found a protective effect of breastfeeding up to 12 months but noted that the association diminishes as duration extends beyond this period.

Conversely, multiple studies indicate that prolonged breastfeeding beyond 24 months increases ECC risk. Chaffee et al. (2014) and Peres et al. (2017) reported a strong link between breastfeeding past 24 months and severe ECC (S-ECC), especially when sugar consumption was high. Abanto et al. (2023) found that while children breastfed between 12 and 24 months had a higher caries risk compared to those breastfed only up to 12 months, this risk was mediated by lower sugar consumption in some cases. Beyond 24 months; however, breastfeeding was associated with ECC regardless of sugar intake.

Nocturnal breastfeeding has also been identified as a contributing factor. Nirunsittirat et al. (2016) and Tham et al. (2015) found that feeding to sleep increased caries risk, reinforcing concerns about reduced salivary flow during sleep. Devenish et al. (2020) further investigated

sustained breastfeeding (≥12 months) and concluded that ECC risk was not solely due to breastfeeding but was significantly influenced by dietary habits, particularly excessive sugar consumption.

Recent clinical trials (Abanto et al. 2023; Devenish et al. 2020; Peres et al. 2017) investigated the impact of sugar in conjunction with breastfeeding as a separate mediating factor. This fact, implies an awareness that most important is the combination of sugar and breast milk, the

frequency and the oral hygiene rather than solely breastfeeding. This is pointed out in the present systematic review as well as at the systematic reviews of Branger et al. (2019); Moynihan et al. (2019) and Moynihan & Kelly (2014).

There is evidence from RCTs that the provision of oral health education to parents/caregivers has a positive impact on ECC risk. A systematic review and meta-analysis from 2019 concluded that there is a 39% reduced risk of ECC prevalence when oral health education was provided to caregivers. However, there was inconsistency amongst the studies (Moynihan et al. 2019). Furthermore, the escalating prevalence of ECC with age may be attributed to an increase in the number of teeth in the oral cavity and the transition from an exclusive milk diet to a mixed diet incorporating solid foods. Beyond the age of two, as children gain autonomy, they tend to opt for unhealthy foods rich in saturated fat and free sugars, contributing to the development of ECC (Manohar et al. 2021). In addition, it has been reported that children who are breastfed for longer durations also have more frequent cariogenic food intakes (Ollila & Larmas 2007).

The findings of the present systematic review are in line with the systematic review of Moynihan et al. (2019). In this systematic review, breastfeeding among other factors that modify the risk of ECC are reported (Moynihan et al. 2019). Regarding the association of breastfeeding and ECC two clinicals studies are reported in this systematic review (Chaffee et al., 2014; Peres et al. 2017). Breastfeeding until the age of 24 months does not have statistically significant impact in ECC in comparison with breastfeeding until the age of 12 months. However, beyond the age of 24 months, breastfeeding is associated with ECC risk. Furthermore, the importance of the diet (sugar intake, carbohydrates) in conjunction with breastfeeding over the age of 12 months is pointed out. Another systematic review by Branger. et al. (2019) reported protecting impact of breastfeeding until the age of 12 months but had difficulty in concluding between protection and aggravation of caries for breastfeeding beyond 12 months because of confounding factors, such as dietary patterns, which vary depending on countries and families, and problems of oral hygiene (Branger et al., 2019).

Branger et al. (2019); Cui et al. (2017); Tham et al. (2015) have indicated in their reviews that breastfeeding for < 12 months has a possible protective effect against dental caries. However, the protective association appears to diminish when breastfeeding extends beyond 12 months, and the current review aligns with these findings. A systematic review published in 2024, (Shrestha et al. 2024) found similar results as above. In addition, breastfeeding for less than 12 or 18 months may incur a protective effect against ECC, although this association seems to dissipate when the breastfeeding duration reaches 24 months. This outcome concurs with the systematic review by Moynihan et al. 2019, reporting that breastfeeding up to 24 months is not associated with a higher risk of ECC (Moynihan et al. 2019).

The strength of the present study when compared to previous sytematic reviews is the inclusion of the highest possible level of evidence studies, prospective cohort studies (n = 7) and RCT (n = 1). In addition, and for first time to our knowledge, the GRADE assessment of the evidence quality was used to strengthen the outcome of the review, regarding the quality of the included studies. There are several limitations of the evidence and methodological challenges which contributes to the conflicting outcomes across studies. Dental caries is a multifactorial disease influenced by host factors, oral bacteria, exposure time, and dietary habits. The escalating prevalence of ECC with age may be attributed to an increase in the number of teeth in the oral cavity and the transition from an exclusive milk diet to a mixed diet incorporating solid foods. Beyond the age of two, as children gain autonomy, they tend to opt for unhealthy foods rich in saturated fat and free sugars, contributing to the development of ECC (Manohar et al. 2021). In addition, it has been reported that children who are breastfed for longer durations also have increased intake of cariogenic food (Ollila & Larmas 2007).

As a result, the complexity of establishing a definite link between breastfeeding and ECC is high due to the confounding factors mentioned above. For this reason, studies have high heterogeneity and high risk of bias. It is thus important for health professionals to focus on the prevention of early childhood caries by promoting early consultation to the dentist, education concerning diet and the use of fluorides. It is clear, however, that breastfeeding is a causative factor for ECC in the age groups above 24 months as seen in this review and previous ones. The impact, however, may be exacerbated by factors such as the consumption of free sugars or can be reduced by oral health education provided to parents/caregivers.

In addition, due to ethical constraints, randomized controlled trials (RCTs) on breastfeeding and ECC cannot be conducted. This means that observational studies, which are prone to bias and confounding variables, form the majority of available evidence. The heterogeneity in study designs, populations, and assessment methods further complicates the ability to draw definitive conclusions. Future research should aim for well-designed prospective cohort studies that account for confounders, such as diet, oral hygiene, and nocturnal feeding

For parents who choose to continue breastfeeding beyond the "safe window" of 12–24 months, preventive measures can significantly reduce ECC risk:

- Early oral hygiene practices: Parents should clean an infant’s gums from birth and introduce toothbrushing as soon as the first tooth erupts.
- Minimizing nocturnal feeding risk: If breastfeeding continues at night beyond 12 months, caregivers should wipe the child’s teeth or offer water afterward to reduce sugar exposure.
- Controlling sugar intake: Parents should limit free sugar consumption, especially in snacks and beverages, to minimize the cariogenic potential of breast milk.
- Early dental visits: A child’s first dental visit should occur by 12 months to establish preventive care and reinforce oral health education.
- Developing good habits in the "shoulder period" (12–24 months): This transition phase is crucial for instilling healthy dietary and hygiene practices before ECC risk escalates.

These recommendations align with public health strategies for ECC prevention and emphasize the importance of educating caregivers rather than discouraging breastfeeding outright. (BSPD, 2024)

### Future research directions

Given the limitations of existing studies, future research should focus on prospective cohort studies that comprehensively track breastfeeding duration, diet, oral hygiene, and other confounders, standardized methods for dietary assessment to control for sugar intake and feeding practices, longitudinal studies examining developmental changes such as tooth eruption, interdental spacing, and salivary composition in relation to ECC risk and interventional studies on oral health education to evaluate its effectiveness in reducing ECC among breastfed children beyond 24 months. While RCTs on breastfeeding duration and ECC are not feasible due to ethical concerns, large-scale, well-controlled observational studies can provide clearer evidence to guide public health recommendations.

## Conclusion

According to the findings of the present systematic review and within its limitations, it has been shown that breastfeeding up to 12 months had a protective impact against Early Childhood Caries (ECC). Breastfeeding for the period 12–23 months was not associated with ECC, although frequent and/or nocturnal breastfeeding mediated by increased sugar consumption had a significant impact on ECC. Breastfeeding over 24 months was associated with an increased prevalence of ECC. These conclusions are based on prospective cohort studies. The role of the dentist/paediatric dentist is crucial and early dental appointments (within the first year of life of the child) should be scheduled to inform parents and reinforce their knowledge regarding dietary habits and feeding practices.

## Supplementary Information

Below is the link to the electronic supplementary material.

Supplementary file1 (DOCX 14 KB)

Supplementary file2 (XLSX 45 KB)

## Data Availability

The data supporting the findings of this study are available in the supplementary material provided with this article.
