# Supplementary material for: Does breastfeeding increase the risk of early childhood caries (ECC)? A systematic review
Source: Eur Arch Paediatr Dent. 2025 May 30;26(4):645–56. doi: 10.1007/s40368-025-01051-4 (PMC12283837; doi:10.1007/s40368-025-01051-4)
Supplement: Supplementary file 1 — Supplementary file1 (DOCX 14 KB) [file 40368_2025_1051_MOESM1_ESM.docx]

| **Πίνακας Στρατηγικής για Συστηματική Ανασκόπηση** | | | | |
| --- | --- | --- | --- | --- |
|  | Electronic databases | Search strategy used | Limitations | Hits |
| 1. | **Medline (Pub Med)**  **17/4/24** | (breastfeeding OR breast feeding) AND milk AND (caries OR decay OR ECC OR early childhood caries) AND (tooth OR teeth) | no | 54 |
| 2. | **Scopus 17/4/24** | (breastfeeding OR breast feeding) AND milk AND (caries OR decay OR ECC OR early childhood caries) AND (tooth OR teeth) | no | 538 |
| 3. | **Science direct 17/4/24** | (breastfeeding OR breast feeding) AND milk AND (caries OR decay OR ECC OR early childhood caries) AND (tooth OR teeth) | no | 1950 |
| 12. | **Google Scholar 17/4/2024** | (breastfeeding OR breast feeding) AND milk AND (caries OR decay OR ECC OR early childhood caries) AND (tooth OR teeth) | Νο | 2360 |
| 13. | **Cochrane Library**  **17/4/24** | (breastfeeding OR breast feeding) AND milk AND (caries OR decay OR ECC OR early childhood caries) AND (tooth OR teeth) | no | 7 |
| Total 4909 | | | | |
